# Machine learning to improve HIV screening using routine data in Kenya

**DOI:** 10.1002/jia2.26436

**Published:** 2025-04-20

**Authors:** Jonathan D. Friedman, Jonathan M. Mwangi, Kennedy J. Muthoka, Benedette A. Otieno, Jacob O. Odhiambo, Frederick O. Miruka, Lilly M. Nyagah, Pascal M. Mwele, Edmon O. Obat, Gonza O. Omoro, Margaret M. Ndisha, Davies O. Kimanga

**Affiliations:** ^1^ Data Science Palladium Group Washington DC USA; ^2^ Division of Global, HIV & TB US Centers for Disease Control and Prevention Nairobi Kenya; ^3^ Kenya Health Information Management Systems (KeHMIS) III Project, Palladium Kenya Nairobi Kenya; ^4^ National AIDS and STIs Control Programme, Ministry of Health Nairobi Kenya; ^5^ USAID United States Agency for International Development, Health Population and Nutrition Office Nairobi Kenya; ^6^ U.S. Department of Defense Nairobi Kenya

**Keywords:** artificial intelligence, electronic health records, HIV infection diagnosis, Kenya, machine learning, routine data

## Abstract

**Introduction:**

Optimal use of HIV testing resources accelerates progress towards ending HIV as a global threat. In Kenya, current testing practices yield a 2.8% positivity rate for new diagnoses reported through the national HIV electronic medical record (EMR) system. Increasingly, researchers have explored the potential for machine learning to improve the identification of people with undiagnosed HIV for referral for HIV testing. However, few studies have used routinely collected programme data as the basis for implementing a real‐time clinical decision support system to improve HIV screening. In this study, we applied machine learning to routine programme data from Kenya's EMR to predict the probability that an individual seeking care is undiagnosed HIV positive and should be prioritized for testing.

**Methods:**

We combined de‐identified individual‐level EMR data from 167,509 individuals without a previous HIV diagnosis who were tested between June and November 2022. We included demographics, clinical histories and HIV‐relevant behavioural practices with open‐source data that describes population‐level behavioural practices as other variables in the model. We used multiple imputations to address high rates of missing data, selecting the optimal technique based on out‐of‐sample error. We generated a stratified 60‐20‐20 train‐validate‐test split to assess model generalizability. We trained four machine learning algorithms including logistic regression, Random Forest, AdaBoost and XGBoost. Models were evaluated using Area Under the Precision‐Recall Curve (AUCPR), a metric that is well‐suited to cases of class imbalance such as this, in which there are far more negative test results than positive.

**Results:**

All model types demonstrated predictive performance on the test set with AUCPR that exceeded the current positivity rate. XGBoost generated the greatest AUCPR, 10.5 times greater than the rate of positive test results.

**Conclusions:**

Our study demonstrated that machine learning applied to routine HIV testing data may be used as a clinical decision support tool to refer persons for HIV testing. The resulting model could be integrated in the screening form of an EMR and used as a real‐time decision support tool to inform testing decisions. Although issues of data quality and missing data remained, these challenges could be addressed using sound data preparation techniques.

## 1 INTRODUCTION

Testing for HIV is essential for linking people living with HIV (PLHIV) to care and treatment services, and persons at risk of HIV acquisition to effective prevention services [1, 2]. This is key to achieving the Joint United Nations Programme on HIV/AIDS (UNAIDS) targets for ending HIV as a global threat by 2030: 95% of PLHIV know their HIV status, 95% of those on treatment and 95% achieving viral suppression [3, 4].

As countries near the first target, identifying undiagnosed PLHIV becomes more challenging. Innovative strategies are needed to identify PLHIV unaware of their status. In Kenya, with HIV prevalence among adults of 3.7% [5], 94% of PLHIV are aware of their HIV status [5], and 2.8% of tests reported through the electronic medical record (EMR) system result in new diagnoses [6]. Current guidelines recommend the use of screening tools to identify persons for testing, including those diagnosed with sexually transmitted infections, those with multiple sexual partners, key populations such as men who have sex with men or female sex workers and those with possible or known HIV exposures, such as sexual or needle‐sharing partner of a person living with HIV or of a person of unknown HIV status. Achieving and maintaining 95% known status requires targeted screening in high‐burden locations and minimizing missed testing opportunities at health facilities [7]. To aid in this, screening tools have been used at health facilities to help identify those who may benefit from HIV testing services, but the majority were developed in high‐income countries or for specific key populations and their predictive accuracy has varied [8, 9, 10].

Several recent studies have explored the potential for machine learning to enhance HIV screening. Machine learning is a subfield of artificial intelligence that uses algorithmic approaches to identify patterns in large datasets that can be used for prediction. Balzer et al. compared the predictive power of model‐based risk scores in predicting HIV seroconversion in rural Uganda and Kenya using data from the Sustainable East Africa Research in Community Health study and found that machine learning improved efficiency in reducing the number of individuals necessary to test to achieve different sensitivity thresholds [11]. Additionally, Mutai et al. applied machine learning to generate and evaluate risk scores using data from Population‐based HIV Impact Assessments (PHIA) and found that socio‐economic variables such as educational attainment and wealth quintile augmented performance [12].

Some studies sought to develop models that emphasized eventual deployment as decision‐support in programmatic settings. Teferi et al. developed a manual risk score generation method that fits on an index card using a form of penalized regression with a small set of predictors and found that the model achieved similar sensitivity to existing screening practices in two regions in Ethiopia with improved specificity [13]. Other models leverage data from EMRs to generate predictions. These models can be integrated in EMRs as real‐time decision support for screening, though their use in low‐and‐middle‐income countries is rare [14, 15].

We aim to develop predictions for HIV test results during the screening process for individuals with no previous HIV diagnosis who seek care at health facilities to inform referral for testing. A machine‐learning‐based screening tool in Kenya could increase yields by screening in undiagnosed positives who are currently screened out, while providing greater flexibility to adjust the overall level of testing to align with resource availability. In our study, we describe the development of a machine learning model to predict an individual's probability of being undiagnosed with HIV using routine HIV testing programme data from Kenya's National Data Warehouse (NDW) and open‐source data.

This activity was reviewed and approved by the U.S. Centers for Disease Control and Prevention (CDC) and was conducted consistent with applicable federal law and CDC policy. §See 45 C.F.R. part 46; 21 C.F.R. part 56.

## 2 METHODS

### 2.1 Data sources

Our dataset consisted of de‐identified individual‐level data from Kenya's NDW collected between April and October 2022 and supplemented with open‐source geospatial datasets from the Institute of Health Metrics and Evaluation (IHME) [16], WorldPop [17] and Meta [18] that describe population‐level HIV‐relevant behavioural norms (see Table 1). Kenya's NDW is a central repository of individual‐level data collected through multiple EMR systems deployed at over 2000 health facilities in Kenya offering HIV prevention and treatment services and transmitted monthly to the warehouse through a data warehouse application programming interface [19]. We included data on those tested, when they were tested, where they were tested and whether the test result was HIV negative or positive. EMR data described the individual‐level information, whereas geospatial data described the geographical characteristics of the location.

**Table 1:** Model features by source, 2022

Feature	Possible values	Missing	Source (year)
Population type	General population, key population, priority population	21%	NDW (2022)
Key population	Female sex worker, men who have sex with men, people who inject with drugs, other, not relevant	21%	NDW
Priority population	Adolescent girls and young women, other, fishermen, not relevant	21%	NDW
Department	Outpatient, voluntary counselling and testing, prevention of mother to child transmission, inpatient, emergency	14%	NDW
Is health worker	No, yes, not relevant	21%	NDW
Sexually active	No, yes, not relevant	53%	NDW
New sexual partner in last 3 months	No, yes, not relevant	55%	NDW
Sexual partner's HIV status	Unknown, negative, positive, not relevant	56%	NDW
Number of partners	Single, multiple, not relevant	56%	NDW
Sex under influence of alcohol	Never, sometimes, always, not relevant	56%	NDW
Unprotected sex for money	No, yes, not relevant	56%	NDW
Condom burst	No, yes, not relevant	56%	NDW
Unprotected sex in last 24 months with partner with unknown HIV status	No, yes, not relevant	56%	NDW
Unprotected sex in last 24 months with partner with known HIV status	No, yes, not relevant	56%	NDW
Pregnant	No, yes, not relevant	12%	NDW
Breastfeeding mother	No, yes, not relevant	15%	NDW
Experienced violence	No, yes	41%	NDW
Ever shared needle while injecting drugs	No, yes, not relevant	20%	NDW
Traditional procedures	No, yes, not relevant	39%	NDW
Mother's HIV status	Positive, negative, unknown, not relevant	3%	NDW
Sex	Female, male	0%	NDW
Marital status	Married, single, minor, divorced, polygamous	18%	NDW
Ever tested for HIV	No, yes	1%	NDW
Months since last test	Last 6 months, 7−12, more than 2 years, 1−2 years, not relevant	22%	NDW
Client tested as	Individual, couple	1%	NDW
Entry point	Outpatient, voluntary counselling and testing, postnatal care, antenatal care, other, paediatric, inpatient, TB, voluntary medical male circumcision	1%	NDW
Test strategy	Hospital patient, non‐hospital patient, index, homebased, other, social network strategy, mobile outreach	0%	NDW
Final test result	Negative, positive	0%	NDW
TB screening outcome	No presumed TB, presumed TB, confirmed TB	4%	NDW
Client self‐tested	No, yes	8%	NDW
Age	Range: [1, 99]	0%	NDW
Day of week of test	Monday, Tuesday, Wednesday, Thursday, Friday, Saturday, Sunday	0%	NDW
Estimated number of births	Range: [0, 26608]	0%	WorldPop (2020)
Estimated number of pregnancies	Range: [0, 37680]	0%	WorldPop
Estimated proportion of female literacy (15−49)	Range: [0.20, 1]	0%	WorldPop
Estimated proportion of people living in poverty	Range: [0.02, 0.85]	0%	WorldPop
Probability of receiving four or more antenatal care visits	Range: [0.35, 0.40]	0%	WorldPop
Probability of receiving postnatal care within 48 hours of delivery	Range: [0.15, 0.22]	0%	WorldPop
Prevalence of skilled birth attendance during delivery	Range: [0.46, 0.74]	0%	WorldPop
Estimated HIV prevalence among adults 15–49	Range: [0.03, 0.31]	0%	IHME (2017)
Estimated number living with HIV adults 15–49	Range: [0.11, 38023]	0%	IHME
Prevalence ever had intercourse young adults	Range: [0.47, 0.84]	0%	IHME
Prevalence of married or living with a partner	Range: [0.50, 0.70]	0%	IHME
Prevalence of male circumcision	Range: [0.35, 1]	0%	IHME
Prevalence of one's current partner living away from home	Range: [0.67, 0.40]	0%	IHME
Prevalence of men with multiple partners in past year	Range: [0.08, 0.26]	0%	IHME
Prevalence of women with multiple partners in past year	Range: [0.01, 0.05]	0%	IHME
Prevalence of self‐reported STI symptoms	Range: [0.03, 0.07]	0%	IHME
Demographic density	Range: [1.92, 101.99]	0%	Meta (2020)

### 2.2 Outcome definition and feature generation

Our modelling objective was to predict the probability an individual was undiagnosed and living with HIV during screening. We included all unique, conclusive HIV test results from individuals who had not been diagnosed with HIV before our study period reported from the NDW. We also obtained 30 attributes associated with the test results, including demographic attributes, clinical history and behavioural practices from the NDW for model features (see Table 1). Geographic attributes from IHME, WorldPop and Meta included prevalence of HIV and associated risk factors, estimates of demographic and social factors, and demographic distributions, respectively. We joined geographic attributes with individual‐level data by health facility, so that individuals at a given facility would have a common vector of geographic attributes, which would be unique for each facility.

We excluded variables that were not properly transmitted to the warehouse due to technical issues. These included whether an individual was experiencing weight loss, lethargy, cough or fever and number of sexual partners. We excluded observations that lacked internal consistency, such as a man or a woman not of child‐bearing age being pregnant at the time of testing. We assigned values of “not relevant” to fields based on known skip logic and responses to prior questions. We converted categorical features into numeric representations, a requirement for some machine learning algorithms.

We considered an approach to missingness as a quasi‐hyperparameter, in which the approach would be determined based on the out‐of‐sample error generated, as with all hyperparameters. First, if the rate of missingness was greater than 50%, we assigned a value of “missing” to the missing observations for that feature rather than impute from observed values. We applied two imputation approaches for the remaining missing values, which affected 18 features. The two approaches included a “simple” approach using mean and mode values for numeric and categorical features, respectively, and a “modelled” approach using Multiple Imputations by Chained Equations [20], in which multiple complete datasets were generated using predictive models and missing values were determined based on the aggregated values generated across imputed datasets.

Thus, we proceeded with three versions of our dataset—no imputation, simple imputation and modelled imputation—and applied all modelling activities described below to each. For the imputed versions of the dataset, we created binary flags as separate features to indicate whether data in the corresponding column was original or imputed. This approach enabled us to preserve insights into the missing data and the potential implications it might carry, as well as maintain a comprehensive dataset.

### 2.3 Model training and evaluation

To complete the preparation for model training, we applied a stratified split of the dataset into 60‐20‐20 proportions, maintaining similar distributions of predictor features and similar positivity rates in each subset [21]. Parameters for imputation for both simple and modelled imputation were calculated using the 60% training dataset only and applied to the 20% validation and 20% test set. This is in line with best practice in machine learning to avoid any information from the validation or test sets leaking into model training and biasing estimates of out‐of‐sample error. For feature selection, we applied the Boruta algorithm on the dataset with simple imputation, an approach that compares the importance of original features with permuted copies of them, which confirmed that all features should be retained for model training [22].

We trained and evaluated four model types including Logistic Regression, Random Forest, AdaBoost and XGBoost. We selected these model types because of the technical feasibility of integration with EMR in offline settings. We conducted a hyperparameter grid search for each tree‐based model, varying dimensions of tree depth and sample size among other hyperparameters, as was relevant to the model type. We repeated the training process for each version of the imputed dataset. For all except XGBoost, we trained separately with simple and modelled imputed datasets. With XGBoost, we trained using the sparse dataset without imputation as well, as the XGBoost algorithm possesses an approach to handling missing data called sparsity‐aware split finding [23].

We evaluated models using Area Under the Precision‐Recall Curve (AUCPR), given the large imbalance in the outcome variable [24]. We also report efficiency and effectiveness metrics. With respect to efficiency for a fixed sensitivity, we considered what proportion of those currently tested would need to be tested to obtain sensitivity rates of 50%, 75% and 95% when sorting individuals by model‐generated risk scores. Programmatically, this measures by how much we could reduce the number of tests performed while still identifying a specified number of individuals likely undiagnosed with HIV. With respect to sensitivity for a fixed proportion target, we considered what recall or sensitivity could be achieved when testing 30%, 50% and 75% of individuals in the dataset regardless of HIV status. Programmatically, this measures what share of undiagnosed individuals we would likely identify when reducing the number of tests performed by a specified amount. We used Shapley Additive Explanations, or SHAP, an approach from the game theory domain designed to quantify the contribution of an individual player to a game's outcome, to identify the importance of features and feature‐value pairs in generating model predictions.

## 3 RESULTS

The dataset consisted of 167,509 HIV tests conducted between June and November 2022. Females represented 64.2% of individuals tested. The average age was 31.1 years. Individuals less than 15 y.o. made up 5.2% of observations. Tests were distributed across the country, coming from 898 health facilities in 37 of Kenya's 47 counties. Tests were concentrated at facilities that had higher rates of adoption of KenyaEMR, through which screening information was collected. The training set included 100,507 observations; validation and test sets included 33,501 observations.

The HIV‐positivity rate in the dataset was 3.4%, which is higher than the rate from testing overall due to under‐reporting of HIV‐negative test results to the NDW based on the review of facilities’ aggregate testing metrics. Positivity rates did not differ substantially by sex (3.4% for females, 3.5% for males). Individuals 15 years of age or under had a lower positivity rate (2.5%) compared to individuals over 15 (3.5%).

### 3.1 AUC‐PR and AUC‐ROC

XGBoost performed best across model types with AUCPR as evaluated on the test set (see Table 2). XGBoost achieved an AUCPR 10.5 times above baseline, which for AUCPR is the prevalence of the positive class, the performance expected from a random model. Although AUCPR is the preferred metric for imbalanced data since it reflects performance only against the positive class of interest, we also report AUC‐ROC. XGBoost achieved an AUC‐ROC of 0.872, compared to a baseline of 0.5, meaning the XGBoost model correctly rates a case as higher risk than a non‐case 87% of the time.

**Table 2:** AUC for HIV testing by model type in Kenya, 2022

Model	Hyperparameters	AUCPR	AUC‐ROC
XGBoost	Tree depth: 8 Subsample ratio: 0.3 Learning rate: 0.1 Learning rounds: 400 Imputation method: Sparse	0.359	0.872
Random Forest	Tree depth: 15 Minimum node size for splitting: 10 Imputation method: Modelled	0.333	0.865
AdaBoost	Tree depth: 6 Learning rounds: 200 Imputation method: Simple	0.252	0.841
Logistic Regression	Imputation method: Simple	0.209	0.814

Sensitivity for a fixed proportion target is the share of all individuals with HIV‐positive test result included in a proportion of individuals tested, sorted by model risk score. Table 3 shows sensitivity for three proportions of observations. XGBoost outperformed other model types at the 75% threshold, but Random Forest performed the best at the 30% and 50% thresholds. With the Random Forest model, we identified 84.6% of all individuals with HIV‐positive test results among the 30% of individuals with the highest risk scores.

**Table 3:** Sensitivity for a fixed proportion target for HIV testing in Kenya, 2022

Model	Percent tested	Sensitivity
XGBoost	30%	84.3%
	50%	92.3%
	75%	98.1%
Random Forest	30%	84.6%
	50%	92.5%
	75%	96.6%
AdaBoost	30%	80.4%
	50%	90.1%
	75%	97.3%
Logistic Regression	30%	75.7%
	50%	87.9%
	75%	96.2%

We considered efficiency for a fixed sensitivity as the share of individuals necessary to test to achieve a given sensitivity. Table 4 shows the percentage tested to achieve a sensitivity of 50%, 75% and 95% for the various machine learning models. The XGBoost model most efficiently reached the 50% sensitivity threshold, requiring testing of only 5.8% of individuals. From a programmatic perspective, it is evident that achieving only a 50% sensitivity level would not be feasible, even if resource conservation is a concern. Stakeholders agreed that evaluation at 95% sensitivity was more applicable, where XGBoost similarly performed best, requiring testing of 58% of individuals.

**Table 4:** Efficiency for a fixed sensitivity for HIV testing in Kenya, 2022

Model	Sensitivity	Percent tested
XGBoost	50%	5.8%
	75%	18.6%
	95%	58%
Random Forest	50%	6.4%
	75%	18.2%
	95%	61.6%
AdaBoost	50%	8.6%
	75%	24.3%
	95%	62.9%
Logistic Regression	50%	10.1%
	75%	29.2%
	95%	68.3%

### 3.2 Results by demographic group

We compared model performance for XGBoost by demographic sub‐group to ascertain if the model would introduce bias against any sub‐group when implemented. For children aged 15 years and under, the model achieved an AUCPR 6.4 times greater than the prevalence of the positive class (0.160/0.025), compared to an AUCPR for adults of 10.9 times the prevalence of the positive class (0.369/0.034). While these ratios are not directly interpretable, the greater multiple achieved for adults indicates the model performed better for that group of individuals. We separately trained a model limiting the training set to children only; however, the multiple generated of 3.6 (0.091/0.025) was lower than that of the training set inclusive of adults. Performance by AUCPR differed marginally by sex. The multiple among males was 10 (0.349/0.035), while that of females was slightly higher at 10.9 (0.366/0.034).

### 3.3 Feature importance

**Figure 1:**
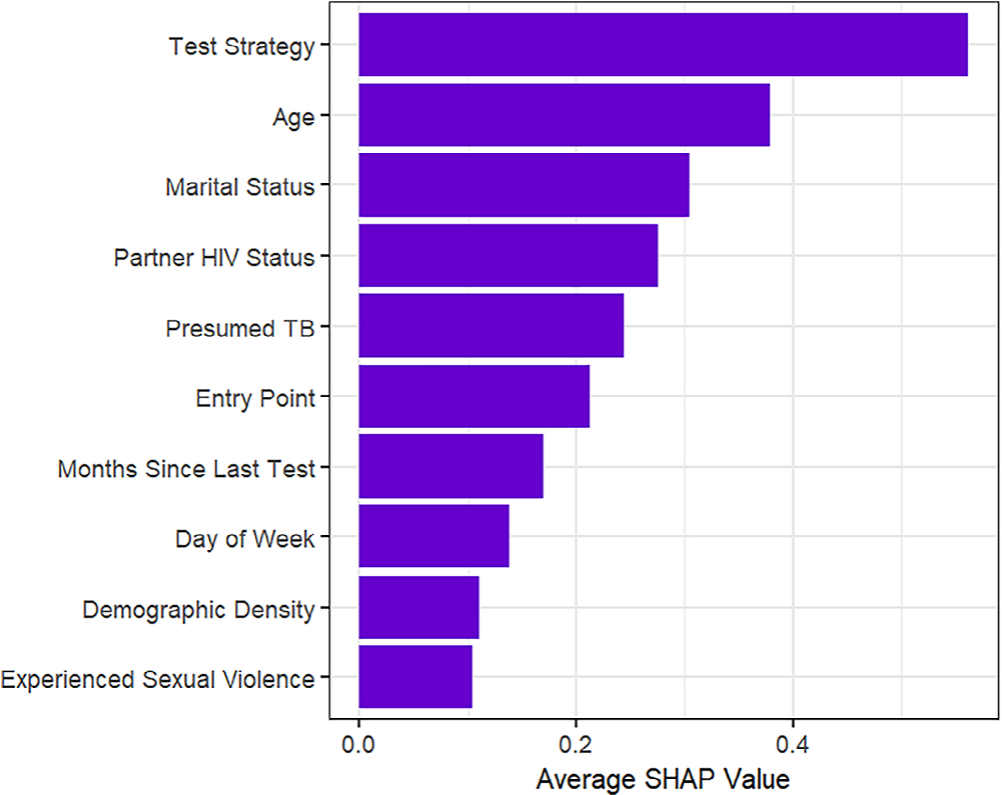
SHAP analysis most important features.

Using SHAP analysis, Figure 1 shows the 10 most important features to the XGBoost model from the original input of 64 predictor variables. The 10 most important features were a variety of feature categories including testing modality (Test Strategy and Entry Point), demographics (Age and Marital Status), clinical history (Presumed TB and Months Since Last Test), sexual history (Partner HIV Status and Experienced Sexual Violence), temporal attributes (Day of Week, representing the day of the week that the test was conducted) and geographic attributes (Demographic Density).

**Figure 2:**
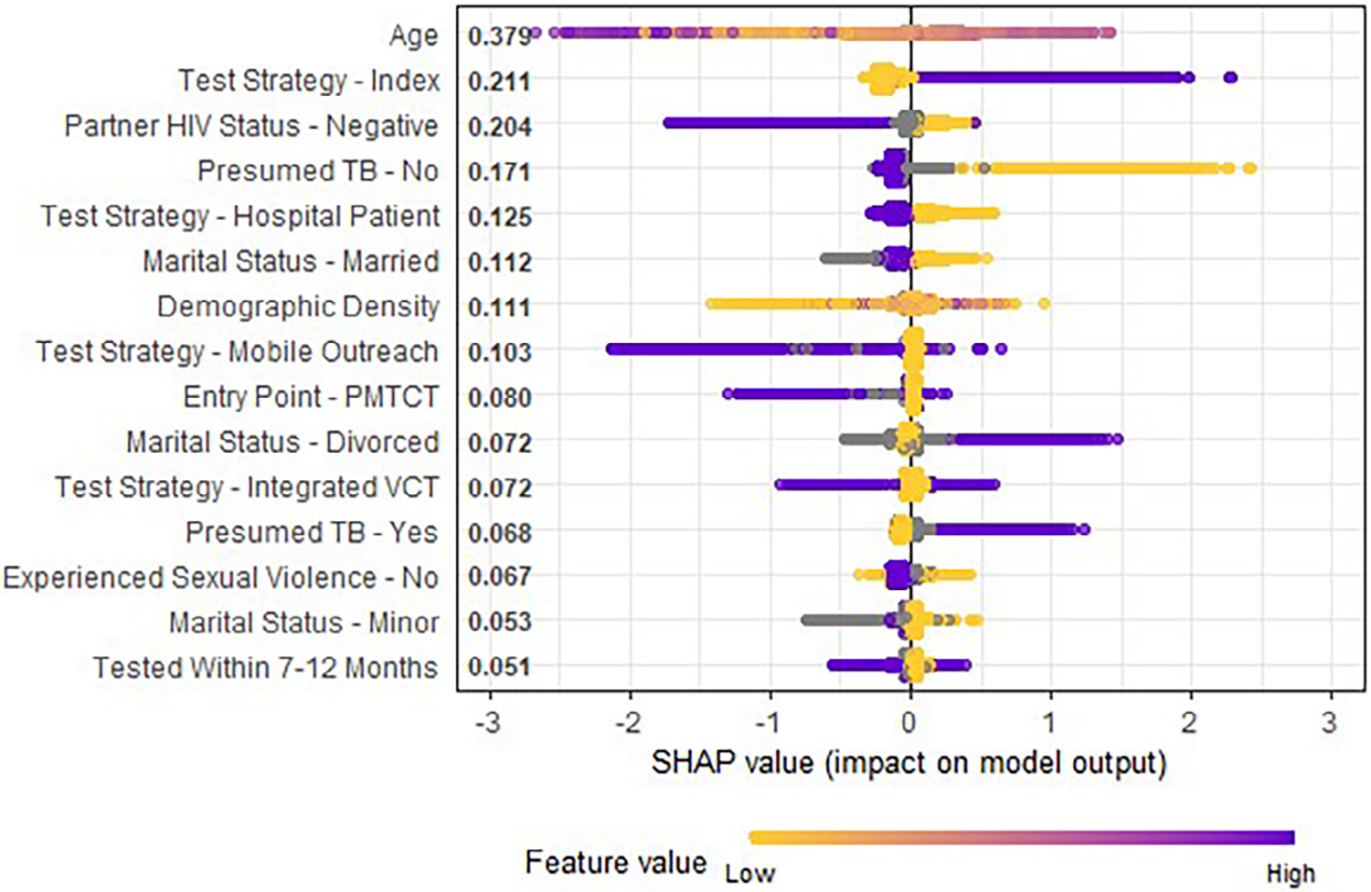
SHAP analysis feature impact.

Apart from identifying important features, SHAP analysis can demonstrate how the XGBoost model used values for each feature to increase or decrease the risk score for a given individual. There were 46 feature‐value pairs across the 10 most important features (e.g. the feature Marital Status contained five values). Figure 2 shows SHAP analysis for the 15 most important feature‐values. The model used feature‐values in purple on the right‐hand side of the graph to suggest an individual was more likely to be undiagnosed with HIV. These included if the individual was part of an index testing strategy, if the individual was divorced and if the individual was presumed to have TB following screening. Feature‐values that suggested an individual was less likely to test positive were those in purple on the left‐hand side of the graph. These included if an individual knew their sexual partner was HIV negative, was married, was presumed to not have TB and had not experienced sexual‐based violence. Feature‐values with purple on both sides played a more intricate role, where their impact on the model's prediction depended on the values of other features.

## 4 DISCUSSION

The model's performance demonstrates its potential utility to better identify individuals who are undiagnosed and living with HIV positive during screening. Although inherent and expected challenges associated with routine programme data such as missingness and data errors existed, these could be resolved during data preparation and as such did not present insurmountable challenges to our goal. Applying different data preparation techniques enabled us to compare the performance of machine learning models trained on sparse and imputed routine program datasets. The XGBoost was the best performing model, its ability to support missing data by default could minimize data preparation efforts in our context. The AUCPR attained by the selected model showed improvement from baseline and compared favourably with other screening tools [8, 9, 10] and similar models trained in research settings where the data collection process for training sets is strictly controlled [11, 12, 13].

The use of geographic population‐level risk behaviours around HIV testing sites together with individual‐specific attributes enabled the model to generate customized predictions for individuals with similar risk factors based on underlying HIV risk associated with the geographic location of HIV testing. The model generated different risk scores for individuals with identical demographic and clinical attributes that sought care in different locations, reflecting the population‐level risk behaviours of the location where they sought care. This approach offers significant advantages in settings like Kenya with highly varying geographical prevalence of HIV.

We observed a better performance for adults compared to children. This was consistent with our hypothesis that there are fewer distinguishing characteristics that apply to children, owing to the lack of relevant behavioural attributes in the dataset, thereby restricting predictive potential. Notably, models achieved a greater performance for children when trained on the entirety of individual data as opposed to training solely on children's data. This may be attributed to two factors. First, models trained on all individual data retain the option to opportunistically split on age, where that is optimal. Second, models trained on children's data alone may miss distinguishing factors that are apparent when combined with adult observations.

There is a good degree of congruence between predictors identified by SHAP and those included in Kenya's testing protocol. The most important predictors from our SHAP analysis included individuals who enter through HIV index testing, which is known to be a high‐yielding HIV testing strategy [25]. Presumed TB infection as a predictor of an HIV‐positive result is consistent with the known association between TB and HIV infections [26]. This is also in line with Kenya's HIV testing recommendations for persons with presumed TB infection. What distinguishes the machine learning approach from existing protocols is the ability to learn complex relationships between these known predictors, as well as the ability to include any number of additional predictors, including from open sources, without adding complexity to clinical workflows.

While the model performed well on historical data, the ability to achieve a programmatic impact by integrating the machine learning model as real‐time decision support is not yet established. There are at least two use cases that could be explored. In one, the model would be used to screen in individuals for testing who otherwise would not be tested, reducing the number of false negatives from current screening practices. In a second, the model would be used to screen out individuals considered low‐risk who otherwise would be tested, excluding categories of individuals for whom testing is mandatory such as those who are pregnant or breastfeeding women, or key populations. The main limiting factor for the former would be the availability of test kits, as the implication is that testing volumes would increase in this scenario. For the latter use case, ethical considerations are paramount due to the prevalence of false negatives, recommending against testing for individuals who are in fact HIV positive. Given the implications, the latter use case should be considered only if a subset of predictions can be defined with an extremely low positivity rate. To properly monitor and assess either use case, feedback mechanisms would need to be established in the EMR in which clinicians indicate reasons for testing so that programmes could observe and assess the impact of model‐driven testing separate from routine testing.

One set of limitations of this study was related to inherent challenges associated with routine collection and transmission of data in clinical settings. Although we resolved most data quality gaps, the baseline and final positivity rates could have been affected by missing records due to unequal data entry of positive and negative cases. Some predictors demonstrated weaker than expected predictive power, particularly those related to unsafe sexual practices, which may result from incomplete or inaccurate responses from individuals provided during screening. Moreover, under‐reporting of HIV test results from facilities that had not adopted EMR limits the generalizability of model results. We urge programmes intending to develop machine learning models in similar contexts to invest in efforts to support complete and accurate data collection and transmission.

## 5 CONCLUSIONS

Our study underscores the practicality of developing predictive models to identify individuals at risk for HIV infection through the utilization of routine programme data. We trained four machine learning models and found the XGBoost model performed best among candidate models, successfully demonstrating the ability to improve screening efficiency and effectiveness by concentrating 95% of individuals with an HIV‐positive test result in the 58% of individuals deemed by the machine learning model to have the greatest probability of testing positive. Effectively utilizing routine data necessitated addressing challenges like missing data and internal inconsistencies, a critical element in working with routine data. We propose to test the use of this model as a real‐time decision support tool to evaluate its applied utility in optimizing testing practices tailored to individual risk profile.

## COMPETING INTERESTS

The authors have no competing interests to declare.

## AUTHORS’ CONTRIBUTIONS

JDF and BAO performed data management, data extraction and data analysis. JDF, JMM, KJM, BAO and JOO participated in the data interpretation. JDF, JMM, KJM and JOO drafted the original manuscript. JDF, JMM, KJM, BAO, JOO, FOM, LMN, PMM, EOO, GOO, MMN and DOK revised all subsequent versions of the manuscript. All authors read and approved the final manuscript.

## FUNDING

Individual‐level data for this analysis was extracted from the National Data Warehouse (NDW) which was supported by the Kenya Health Management Information Systems, a project managed by Palladium Kenya with oversight from the National AIDS and STIs Control Programme (NASCOP), Ministry of Health. Palladium Kenya is an implementing partner funded by PEPFAR through the CDC Kenya to technically support MOH in the provision of strategic information and digital health solutions. Palladium's scope of work includes strengthening data systems, analytics and AI/ML solutions to enhance HIV programming. Palladium as a technical partner does not inherently introduce bias in this study. Palladium's role is to provide technical support for data systems, analytics and capacity‐building for MOH staff. All data processes adhered to national guidelines, with oversight and collaboration from MOH and relevant stakeholders to ensure data integrity and neutrality in the analyses.

## PEPFAR ATTRIBUTION OF SUPPORT

This publication has been supported by the President's Emergency Plan for AIDS Relief (PEPFAR) through the Centers for Disease Control and Prevention (CDC) under the terms of 1U2GGH001226.

## CDC AUTHORSHIP DISCLAIMER

The findings and conclusions in this report are those of the author(s) and do not necessarily represent the official position of the funding agencies.

## Data Availability

The data that support the findings of this study are available on request from the corresponding author. The data are not publicly available due to privacy or ethical restrictions. The study was based on de‐identified data on humans and did not involve experiments on animal or human tissues. The individual‐level data was obtained as part of routine HIV testing through multiple EMR systems and uploaded to the National Data Warehouse in Kenya. The research was carried out in accordance with the provisions of the relevant laws, policies and regulations as set out by the Research and Ethics Committee of Jomo Kenyatta University of Agriculture and Technology (JKU/ISERC/02316/0880), and the research license provided by the National Commission for Science, Technology & Innovation (NACOSTI/P/23/27455). This activity was reviewed by CDC, deemed not research, and was conducted consistent with applicable federal law and CDC policy. (§See e.g. 45 C.F.R. part 46, 21 C.F.R. part 56; 42 U.S.C. §241(d); 5 U.S.C. §552a; 44 U.S.C. §3501 et seq.) These approvals granted a waiver of individual consent as the study involved secondary analysis of de‐identified routine programme data. A hashing algorithm was applied to ensure full anonymization before extraction of the datasets and the investigators did not interact with the study subjects.
